# Development and the need for implementation of a health-related quality of life measurement strategy for patients with soft tissue sarcoma undergoing preoperative radiotherapy

**DOI:** 10.2340/1651-226X.2025.43110

**Published:** 2025-04-30

**Authors:** Geraldien F. Foppele, Lisette M. Wiltink, Marta Fiocco, Jules Lansu, Astrid N. Scholten, Winan J. van Houdt, Yvonne Schrage, Winette T. A van der Graaf, Rick L. M. Haas, Olga Husson

**Affiliations:** a Department of Radiotherapy, Netherlands Cancer Institute, Amsterdam, The Netherlands; b Department of Radiotherapy, Leiden University Medical Centre, Leiden, The Netherlands; c Mathematical Institute, University of Leiden, Leiden, The Netherlands; d Department of Biomedical Data Sciences, Leiden University Medical Centre, Leiden, The Netherlands; e Princess Máxima Centre for Paediatric Oncology, Utrecht, The Netherlands; f Department of Radiotherapy, Haga Hospital, The Hague, The Netherlands; g Department of Surgery, Netherlands Cancer Institute, Amsterdam, The Netherlands; h Department of Medical Oncology, Netherlands Cancer Institute, Amsterdam, The Netherlands; i Department of Medical Oncology, Erasmus MC Cancer Institute, Erasmus Medical Centre, Rotterdam, The Netherlands; j Department of Public Health & Surgical Oncology, Erasmus Medical University Centre, Rotterdam, The Netherlands

**Keywords:** Soft tissue sarcoma, health-related quality of life, preoperative radiotherapy, hypofractionated radiotherapy, sarcoma-specific questionnaire, SCOPES trial

## Abstract

**Background and purpose:**

Soft tissue sarcomas (STS) are a rare and heterogeneous group of tumours. Treatment strategies are based on histological subtype, patient performance status, tumour location, and size. Common treatment modalities include surgery, (neo)adjuvant chemotherapy, and (neo)adjuvant radiotherapy. While disease control and survival remain primary research focuses, health-related quality of life (HRQoL) is equally important. Accurately assessing HRQoL outcomes is particularly difficult due to the heterogeneity of STS, variations in treatment, tumour location and surgical interventions, and the prevalence of these rare cancers.

**Patients and methods:**

To address this gap, a sarcoma-specific tool was developed complementing the most used cancer-generic EORTC QLQ-C30 questionnaire, with additional items tailored to the specific challenges of radiotherapy and surgery in sarcoma patients. The final questionnaire consists of the EQ-5D-5L, EORTC QLQ-C30, and additional EORTC Item Library items addressing stiffness, pain in muscles and bones, scar pain, as well as items of the PRO-CTCAE. The selection was made with significant input from patients with STS who underwent radiotherapy previously and with input from clinicians who were present at the multidisciplinary consultation.

**Results and Interpretation:**

The newly developed HRQoL tool is currently undergoing validation within the SCOPES trial (NTC04425967), evaluating HRQoL outcomes in patients undergoing standard versus hypofractionated radiotherapy. This tool will lead to better understanding the full impact of treatment on sarcoma patients and hopefully, ultimately improve their HRQoL. Moreover, it provides a standardized framework that can be utilized across studies, facilitating the comparison of HRQoL data and enabling more consistent and comprehensive insights into patient outcomes.

Soft tissue sarcomas (STS) comprise a rare and heterogeneous group of tumours, with an incidence of 800 to 900 cases annually in the Netherlands [1]. Treatment strategies are based on histological subtype, patient performance status, tumour location, and size. Common treatment modalities include surgery, (neo)adjuvant chemotherapy, and (neo)adjuvant radiotherapy. Internationally, the standard regimen for preoperative radiotherapy is 25 fractions of 2 Gy [2–6]. More recently, studies have explored different fractionation schedules and doses in the context of preoperative radiotherapy [7–14]. The ongoing randomised SCOPES trial (short course of preoperative radiotherapy in head and neck-, trunk- and extremity STS; NTC04425967) investigates 14 × 3 Gy compared to 25 × 2 Gy. The trial is hypothesised to offer favourable outcomes and health-related quality of life (HRQoL) after 14 × 3Gy.

Current research and clinical trials primarily focus on tumour control and patient survival. Nonetheless, while disease outcomes are crucial, it is equally important to focus on HRQoL, as patients must live with the enduring consequences of their condition. While advancements in STS treatment, such as hypofractionated radiotherapy, are promising, the lack of a sarcoma-specific HRQoL questionnaire presents a significant challenge. Accurately assessing HRQoL outcomes is particularly difficult due to the heterogeneity of STS, variations in treatment, tumour location and surgical interventions, and the prevalence of these rare cancers [15–17].

Health-related quality of life in patients with STS is largely influenced by the tumour location and the treatment. Therefore, research often employs a combination of generic or cancer-specific HRQoL questionnaires along with tumour-specific tools. Such tools will allow for a more precise assessment of the impact of modified radiotherapy regimens on patients’ overall well-being, ensuring that treatment benefits are aligned with patient experiences and preferences [18]. However, a dedicated tool for assessing HRQoL in patients with STS undergoing preoperative radiotherapy is currently lacking. Ongoing trials aiming at the development of a questionnaire are time-consuming due to the need to interview multiple patients and specialists across various centres. As research into radiotherapy has significantly increased in recent years, the demand for such questionnaires is high. To address this need, a sarcoma-specific radiotherapy tool was developed complementing the most used cancer-generic EORTC QLQ-C30 questionnaire, with additional items tailored to the specific challenges of radiotherapy in sarcoma patients. This study specifically focuses on adult patients with STS who received preoperative radiotherapy as part of a neo-adjuvant treatment in a curative setting. The influence of neo-adjuvant chemotherapy on HRQoL is associated with a different profile, and therefore, chemotherapy is not within the scope of this HRQoL tool.

The development process involved a comprehensive review of existing HRQoL measures for cancer, followed by the narrow-down selection of treatment-specific questions derived from the EORTC Item Library and PRO-CTCAE, consisting of all validated items of EORTC QLQ questionnaires [19]. The selection was made with significant input from patients with STS who underwent radiotherapy previously and with input from clinicians who were present at the multidisciplinary consultation. Initially, five patients were interviewed using open-ended questions to explore their experiences and concerns regarding treatment. Following these interviews, patients were presented with various potential questionnaire items, which they then ranked in terms of relevance and importance. Additionally, patients were asked to provide feedback on the length of the questionnaire to ensure it was not perceived as too long. This patient-centred approach ensured that the final item list is both comprehensive and sensitive to the specific needs of sarcoma patients (Figure 1). The final questionnaire (Supplementary Appendix 1) after selection consists of the EQ-5D-5L, EORTC QLQ-C30, and additional EORTC Item Library items addressing stiffness, pain in muscles and bones, scar pain, as well as items of the PRO-CTCAE, forming a combination of a generic HRQoL questionnaire (EORTC QLQ-C30) and items focused on extremity and trunk (EORTC Item Library and PRO-CTCAE items). Items of the PRO-CTAE were items on decreased appetite, nausea, constipation, diarrhoea, swelling, rash, skin dryness, itching, radiation skin reaction, skin darkening, concentration, memory, insomnia, fatigue, anxiousness, discouragement, and sadness. In addition three options were left open in which patients could enter an adverse event/symptom not assessed [20]. The newly developed HRQoL tool is currently undergoing validation within the SCOPES trial.

**Figure 1:**
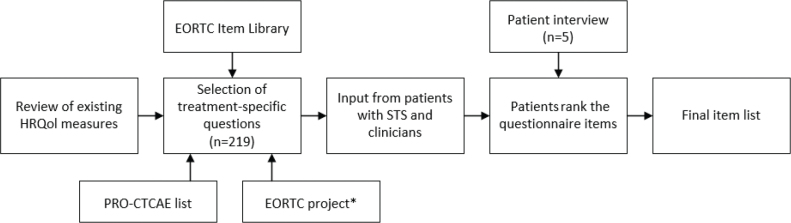
Flowchart illustrating the development of the final item list for patients with soft tissue sarcoma undergoing preoperative radiotherapy. *Items available at that time from the EORTC project by D. Den Hollander et al. [18].

The newly developed soft tissue sarcoma-specific radiotherapy item list offers a valuable tool for both clinical practice and research. For clinical practice, it is advised to administer the item list at baseline (T1), at the peak of toxicity at the end of neo-adjuvant radiotherapy (T2), just before surgery (T3), 30 days post-surgery (T4) and at the end of each follow-up year (T5), as currently used in the SCOPES trial. It provides a way to accurately measure the impact of different radiotherapy regimens on patients’ HRQoL, allowing for more informed treatment decisions and is currently used in the SCOPES trial to compare patients’ outcomes in both arms. Moreover, this questionnaire can serve as a model for developing similar tools for other rare and complex diseases, where generic QoL questionnaires may not be sufficient, but validated questionnaires are hard to develop given the limited patient numbers. This questionnaire provides a framework for how a tool can be developed within a short timeframe.

In conclusion, the creation of a soft tissue sarcoma-specific radiotherapy HRQoL questionnaire for patients treated with radiotherapy fills a critical gap in HRQoL assessment. This tool will enable radiation oncologists to better understand the full impact of treatment on sarcoma patients and hopefully, ultimately improve their HRQoL. Moreover, it provides a standardised framework that can be utilised across studies, facilitating the comparison of HRQoL data and enabling more consistent and comprehensive insights into patient outcomes.

## Supplementary Material

Development and the need for implementation of a health-related quality of life measurement strategy for patients with soft tissue sarcoma undergoing preoperative radiotherapy

## Data Availability

The data supporting the development of the soft tissue sarcoma-specific radiotherapy HRQoL tool is not publicly available due to privacy concerns and the sensitive nature of patient interviews. The tool itself, including the final item list, is included in Supplementary Appendix 1 and is currently being validated within the SCOPES trial (NTC04425967).

## Highlights

- A health-related quality of life measurement strategy was developed for patients with soft tissue sarcoma undergoing preoperative radiotherapy.
- The questionnaire assesses patient outcomes and is implemented in the SCOPES trial.
- This tool sets a standard for HRQoL evaluation in rare, complex diseases like sarcomas.
