# Supplementary material for: Development and the need for implementation of a health-related quality of life measurement strategy for patients with soft tissue sarcoma undergoing preoperative radiotherapy
Source: Acta Oncol. 2025 Apr 30;64:43110. doi: 10.2340/1651-226X.2025.43110 (PMC12053518; doi:10.2340/1651-226X.2025.43110)
Supplement: Development and the need for implementation of a health-related quality of life measurement strategy for patients with soft tissue sarcoma undergoing preoperative radiotherapy [file AO-64-43110-s1.pdf]

EQ5D-5L

Under each heading, please tick the **one** box that best describes your health **today**.

A. Mobility

I have no problems in walking about ❒
I have slight problems in walking about ❒
I have moderate problems in walking about ❒
I have severe problems in walking about ❒
I am unable to walk about ❒

B. Self-care

I have no problems washing or dressing myself ❒
I have slight problems washing or dressing myself ❒
I have moderate problems washing or dressing myself. ❒
I have severe problems washing or dressing myself ❒
I am unable to wash or dress myself ❒

C. Usual activities (e.g. work, study, housework, family or leisure activities)

I have no problems doing my usual activities ❒
I have slight problems doing my usual activities ❒
I have moderate problems doing my usual activities ❒
I have severe problems doing my usual activities ❒
I am unable to do my usual activities ❒

D. Pain/discomfort

I have no pain or discomfort ❒
I have slight pain or discomfort ❒
I have moderate pain or discomfort ❒
I have severe pain or discomfort ❒
I have extreme pain or discomfort ❒

E. Anxiety/depression

I am not anxious or depressed ❒
I am slightly anxious or depressed ❒
I am moderately anxious or depressed ❒
I am severely anxious or depressed ❒
I am extremely anxious or depressed ❒

—

- We would like to know how good or bad your health is **today**
- This scale is numbered from 0 to 100
- 100 means the **best** health you can imagine
  0 means the **worst** health you can imagine

- Mark an X on the scale to indicate how your health is TODAY
- Now, please write the number you marked on the scale in the box below

YOUR HEALTH TODAY =

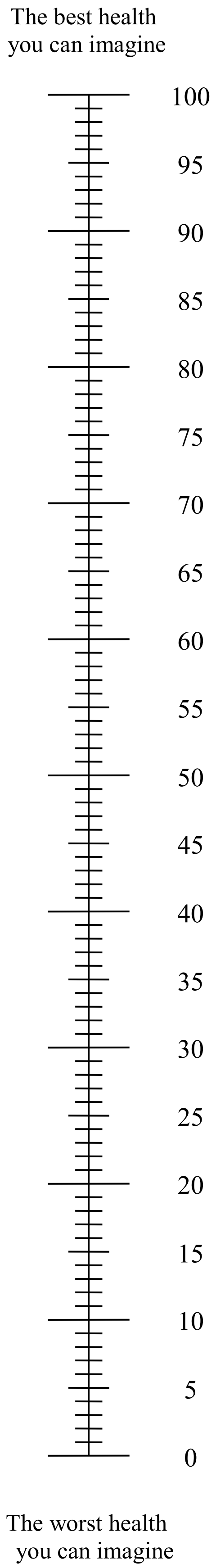

EORTC-QLQ-C30

Your health
We are interested in your health-related quality of life. Please answer all the questions below choosing the answer that is most applicable to you.

**Not** time-specific questions		**Not at all**	**A little**	**Quite a bit**	**Very much**
1.	Do you have any trouble doing strenuous activities like carrying a heavy shopping bag or a suitcase?	1 ❑	2 ❑	3 ❑	4 ❑
2.	Do you have any trouble taking a long walk?	1 ❑	2 ❑	3 ❑	4 ❑
3.	Do you have any trouble taking a short walk outside of the house?	1 ❑	2 ❑	3 ❑	4 ❑
4.	Do you need to stay in bed or a chair during the day?	1 ❑	2 ❑	3 ❑	4 ❑
5.	Do you need help with eating, dressing, washing yourself or using the toilet?	1 ❑	2 ❑	3 ❑	4 ❑

During the past **week (last seven days):**		**Not at all**	**A little**	**Quite a bit**	**Very much**
6.	Were you limited in doing either your work or other daily activities?	1 ❑	2 ❑	3 ❑	4 ❑
7.	Were you limited in pursuing your hobbies or other leisure time activities?	1 ❑	2 ❑	3 ❑	4 ❑
8.	Were you short of breath?	1 ❑	2 ❑	3 ❑	4 ❑
9.	Have you had pain?	1 ❑	2 ❑	3 ❑	4 ❑
10.	Did you need to rest?	1 ❑	2 ❑	3 ❑	4 ❑
11.	Have you had trouble sleeping?	1 ❑	2 ❑	3 ❑	4 ❑
12.	Have you felt weak?	1 ❑	2 ❑	3 ❑	4 ❑
13.	Have you lacked appetite?	1 ❑	2 ❑	3 ❑	4 ❑
14.	Have you felt nauseated?	1 ❑	2 ❑	3 ❑	4 ❑
15.	Have you vomited?	1 ❑	2 ❑	3 ❑	4 ❑
16.	Have you been constipated?	1 ❑	2 ❑	3 ❑	4 ❑
17.	Have you had diarrhea?	1 ❑	2 ❑	3 ❑	4 ❑

18.	Were you tired?	1 ❑	2 ❑	3 ❑	4 ❑
19.	Did pain interfere with your daily activities?	1 ❑	2 ❑	3 ❑	4 ❑
20.	Have you had difficulty in concentrating on things, like reading a newspaper or watching television?	1 ❑	2 ❑	3 ❑	4 ❑
21.	Did you feel tense?	1 ❑	2 ❑	3 ❑	4 ❑
22.	Did you worry?	1 ❑	2 ❑	3 ❑	4 ❑
23.	Did you feel irritable?	1 ❑	2 ❑	3 ❑	4 ❑
24.	Did you feel depressed?	1 ❑	2 ❑	3 ❑	4 ❑
25.	Have you had difficulty remembering things?	1 ❑	2 ❑	3 ❑	4 ❑
26.	Has your physical condition or medical treatment interfered with your family life?	1 ❑	2 ❑	3 ❑	4 ❑
27.	Has your physical condition or medical treatment interfered with your social activities?	1 ❑	2 ❑	3 ❑	4 ❑
28.	Has your physical condition or medical treatment caused you financial difficulties?	1 ❑	2 ❑	3 ❑	4 ❑

29. How would you rate your overall health during the past week?

Very poor Excellent

1❑ 2 ❑ 3 ❑ 4 ❑ 5 ❑ 6 ❑ 7❑

30. How would you rate your overall quality of life during the past week?

Very poor Excellent

1❑ 2 ❑ 3 ❑ 4 ❑ 5 ❑ 6 ❑ 7❑

Sometimes patients report having the following symptoms. Please answer all the questions below choosing the answer that is most applicable to you.

During the past **week (last seven days):**		**Not at all**	**A little**	**Quite a bit**	**Very much**
31.	Did you experience stiffness in your joints?	1 ❑	2 ❑	3 ❑	4 ❑
32.	Did you have any pain in muscles or bones?	1 ❑	2 ❑	3 ❑	4 ❑
33.	Did your scar interfere with your daily activities?	1 ❑	2 ❑	3 ❑	4 ❑

PRO-CTCAE

As individuals go through treatment for their cancer they sometimes experience different symptoms and side effects. For each question, please select the one response that best describes your experience over the past 7 days.

Decreased appetite

In the last 7 days, what was the **severity** of your **decreased appetite** at its **worst?**

❑ None ❑ Mild ❑ Moderate ❑Severe ❑ Very severe

In the last 7 days, how much did **decreased appetite interfere** with your usual or daily activities?

❑ None ❑ Mild ❑ Moderate ❑Severe ❑ Very severe

Nausea

In the last 7 days, how **often** did you have **nausea?**

❑ None ❑ Mild ❑ Moderate ❑Severe ❑ Very severe

In the last 7 days, what was the **severity** of your **nausea** at its worst?

❑ None ❑ Mild ❑ Moderate ❑Severe ❑ Very severe

Constipation

In the last 7 days, what was the **severity** of your constipation at its worst?

❑ None ❑ Mild ❑ Moderate ❑Severe ❑ Very severe

Diarrhea

In the last 7 days, how **often** did you have **loose or watery stools (diarrhea/diarrhoea)?**

❑ None ❑ Mild ❑ Moderate ❑Severe ❑ Very severe

Swelling

In the last 7 days, how **often** did you have **arm or leg swelling?**

❑ None ❑ Mild ❑ Moderate ❑Severe ❑ Very severe

In the last 7 days, what was the **severity** of your **arm and leg swelling** at its **worst?**

❑ None ❑ Mild ❑ Moderate ❑Severe ❑ Very severe

In the last 7 days, how much did **arm and leg swelling interfere** with you usual or daily activities?

❑ None ❑ Mild ❑ Moderate ❑Severe ❑ Very severe

Muscle pain

In the last 7 days, how **often** did you have **aching muscles?**

❑ None ❑ Mild ❑ Moderate ❑Severe ❑ Very severe

In the last 7 days, what was the **severity** of your **aching muscles** at its **worst?**

❑ None ❑ Mild ❑ Moderate ❑Severe ❑ Very severe

In the last 7 days, how much did **aching muscles interfere** with your usual or daily activities?

❑ None ❑ Mild ❑ Moderate ❑Severe ❑ Very severe

Joint pain

In the last 7 days, how **often** did you have **aching joints (such as elbows, knees, shoulders)?**

❑ None ❑ Mild ❑ Moderate ❑Severe ❑ Very severe

In the last 7 days, what was the **severity** of your **aching joints (such as elbows, knees, shoulders)** at their **worst?**

❑ None ❑ Mild ❑ Moderate ❑Severe ❑ Very severe

In the last seven days, how much did **aching joints (such as elbows, knees, shoulders) interfere** with your usual or daily activities?

❑ None ❑ Mild ❑ Moderate ❑Severe ❑ Very severe

Rash

In the last 7 days, did you have any **rash?**

❑ Yes ❑ No

Skin dryness

In the last.7 days, what was the **severity** of your **dry skin** at its **worst?**

❑ None ❑ Mild ❑ Moderate ❑Severe ❑ Very severe

Itching

In the last 7 days, what was the **severity** of your **itchy skin** at its **worst?**

❑ None ❑ Mild ❑ Moderate ❑Severe ❑ Very severe

Radiation skin reaction

In the last 7 days, what was the **severity** of your **skin burns from radiation** at its **worst?**

❑ None ❑ Mild ❑ Moderate ❑Severe ❑ Very severe

Skin darkening

In the last 7 days, did you have any **unusual darkening of the skin?**

❑ Yes ❑ No

Concentration

In the last 7 days, what was the **severity** of your **problems with concentration** at its **worst?**

❑ None ❑ Mild ❑ Moderate ❑Severe ❑ Very severe

In the last 7 days, how much did **problems with concentration interfere** with your usual or daily activities?

❑ None ❑ Mild ❑ Moderate ❑Severe ❑ Very severe

Memory

In the last 7 days, what was the **severity** of your **problems with memory** at their **worst?**

❑ None ❑ Mild ❑ Moderate ❑Severe ❑ Very severe

In the last 7 days, how much did **problems with memory interfere** with your usual or daily activities?

❑ None ❑ Mild ❑ Moderate ❑Severe ❑ Very severe

Insomnia

In the last 7 days, what was the **severity** of your **insomnia (including difficulty falling asleep, staying asleep or waking up early)** at its **worst?**

❑ None ❑ Mild ❑ Moderate ❑Severe ❑ Very severe

In the last 7 days, how much did **insomnia (including difficulty falling asleep, staying asleep or waking up early) interfere** with your usual or daily activities?

❑ None ❑ Mild ❑ Moderate ❑Severe ❑ Very severe

Fatigue

In the last 7 days, what was the **severity** of your **fatigue, tiredness or lack of energy** at its **worst?**

❑ None ❑ Mild ❑ Moderate ❑Severe ❑ Very severe

In the last 7 days, how much did **fatigue, tiredness or lack of energy interfere** with your usual or daily activities?

❑ None ❑ Mild ❑ Moderate ❑Severe ❑ Very severe

Anxious

In the last 7 days, how **often** did you feel **anxious?**

❑ None ❑ Mild ❑ Moderate ❑Severe ❑ Very severe

In the last 7 days, what was the **severity** of your **anxiety** at its **worst?**

❑ None ❑ Mild ❑ Moderate ❑Severe ❑ Very severe

in the last 7 days, how much did **anxiety interfere** with your usual or daily activities?

❑ None ❑ Mild ❑ Moderate ❑Severe ❑ Very severe

Discouraged

In the last 7 days, how **often** did you feel that **nothing could cheer you up?**

❑ None ❑ Mild ❑ Moderate ❑Severe ❑ Very severe

In the last 7 days, what was the **severity** of your **feelings that nothing could cheer you up** at their **worst?**

❑ None ❑ Mild ❑ Moderate ❑Severe ❑ Very severe

In the last 7 days, how much did **feeling that nothing could cheer you up interfere** with your usual or daily activities?

❑ None ❑ Mild ❑ Moderate ❑Severe ❑ Very severe

Sad

In the last 7 days, how **often** did you have **sad or unhappy feelings?**

❑ None ❑ Mild ❑ Moderate ❑Severe ❑ Very severe

In the last 7 days, what was the **severity** of your **sad or unhappy feelings** at their **worst?**

❑ None ❑ Mild ❑ Moderate ❑Severe ❑ Very severe

In the last 7 days, how much did **sad or unhappy feeling interfere** with your usual or daily activities?

❑ None ❑ Mild ❑ Moderate ❑Severe ❑ Very severe

Other symptoms
Do you have other symptoms that you wish to report?

❑ Yes ❑ No

Please list any other symptoms:

1: ……………………………………………………….

In the last 7 days, what was the **severity** of this symptom at its **worst?**

❑ None ❑ Mild ❑ Moderate ❑Severe ❑ Very severe

2: ……………………………………………………….

In the last 7 days, what was the **severity** of this symptom at its **worst?**

❑ None ❑ Mild ❑ Moderate ❑Severe ❑ Very severe

3: ……………………………………………………….

In the last 7 days, what was the **severity** of this symptom at its **worst?**

❑ None ❑ Mild ❑ Moderate ❑Severe ❑ Very severe

4: ……………………………………………………….

In the last 7 days, what was the **severity** of this symptom at its **worst?**

❑ None ❑ Mild ❑ Moderate ❑Severe ❑ Very severe

5: ……………………………………………………….

In the last 7 days, what was the **severity** of this symptom at its **worst?**

❑ None ❑ Mild ❑ Moderate ❑Severe ❑ Very severe
